# Antiretroviral generics

**DOI:** 10.1186/1742-4690-9-S1-I20

**Published:** 2012-05-25

**Authors:** Véronique Andrieu

**Affiliations:** 1 Galenic Laboratory Faculty of Pharmacy, Marseille, France

## 

The current definition for generic medicinal products is found in Directive 2001/83/EC, Article 10(2)(b), which states that a generic medicinal product is a product which has the **same qualitative and quantitative composition** in active substances and the same pharmaceutical form as the reference medicinal product, and whose **bioequivalence** with the reference medicinal product has been demonstrated by appropriate bioavailability studies.

The **different salts, esters, ethers, isomers, mixtures of isomers, complexes or derivatives** of an active substance are considered to be the same active substance, unless they differ significantly in properties with regard to safety and/or efficacy.

Furthermore, the various immediate-release oral pharmaceutical forms shall be considered to be one and the same pharmaceutical form.

Concept of bioequivalence is fundamental: The purpose of establishing bioequivalence is to demonstrate equivalence in biopharmaceutics quality between the generic medicinal product and a reference medicinal product in order to allow **bridging of preclinical tests and of clinical trials** associated with the reference medicinal product.

In bioequivalence studies, the plasma concentration time curve is generally used to assess the rate and extent of absorption. Selected pharmacokinetic parameters and preset acceptance limits allow the final decision on bioequivalence of the tested products.

AUC, the area under the concentration time curve, reflects the extent of exposure. Cmax, the maximum plasma concentration or peak exposure, and the time to maximum plasma concentration, tmax, are parameters that are influenced by absorption rate.

In studies to determine bioequivalence after a single dose, the parameters to be analysed are AUC(0-t), or, when relevant, AUC(0-72h), and Cmax. For these parameters the 90% confidence interval for the ratio of the test and reference products should be contained within the acceptance interval of 80.00-125.00%. To be inside the acceptance interval the lower bound should be ≥ 80.00% when rounded to two decimal places and the upper bound should be ≤ 125.00% when rounded to two decimal places.

Evaluation of Generic medicinal product is also based on the pharmaceutical quality of the active substance: absence of impurities,.. and of the finished product : reproducibility of the manufacturing process, stability,….

A table of the Antiretroviral drugs and of the Generics available in Europe is presented.

